# An Ethics Toolkit to Support Animal-Centered Research and Design

**DOI:** 10.3389/fvets.2022.891493

**Published:** 2022-05-10

**Authors:** Luisa Ruge, Clara Mancini

**Affiliations:** School of Computing and Communications, The Open University, Milton Keynes, United Kingdom

**Keywords:** ethics toolkit, animal ethics and welfare, animal-computer interaction, animal-centered design, animal research, research practice

## Abstract

Designers and researchers who work with animals need to employ an array of ethical competencies to guarantee the welfare of animals taking part in animal-centered research. The emerging field of Animal-Computer Interaction (ACI), which deals with the design of animal-centered interactive systems, considers ethics a fundamental concern when working with animals, and ACI researchers have proposed ethics frameworks in response to these concerns. Ethical approaches proposed within the field tend to be normative but, on their own, norms may not be sufficient to support designers who will inevitably face unexpected and ethically charged situations as the research progresses. During a research project, focused on the design of dog-friendly controls for Mobility Assistance Dogs (MADs), these limitations came to the fore. Drawing from situated ethics approaches, developed to support researchers' ethical engagement with vulnerable populations such as children and differently abled adults, this paper presents an ethics toolkit that aims to support animal-centered research and design by enabling researchers to make ethically sound situated decisions as their work progresses. The toolkit comprises three templates, each of which asks a series of questions aiming to articulate the ethical baselines of individual team members and of their research project, and to inform the development of a series of ethical guiding statements to better prepare designers to make ethical situated decisions. The application of the toolkit during the research with MADs helped the field researcher to clearly and systematically articulate the project's ethos and understand the ethical stance that guided the research team's interactions with the dogs, their trainers, and their human partners throughout the project. It also fostered a practice of active reflection within the team, which helped them to maintain their commitment to the project's ethos in the face of unexpected ethical challenges. We propose that, beyond supporting ACI research, the toolkit could support the ethical engagement of researchers and practitioners who work for and with animals in many other settings.

## Introduction

Ethics is a fundamental concern for disciplines that work with animals, particularly when the aim is to study and design animal-centered interactive systems. Extending the scope of the human-centered disciplines of Human-Computer Interaction (1) and Interaction Design (2), Animal-Computer Interaction (ACI) (3) investigates how animals interact with technology and how animal-centered interactions can be designed. This is arguably more important now than ever before, given animals' increasing exposure to technology.

Most ethical approaches proposed within the field of ACI have been normative in nature, providing researchers with principles to orient their understanding of what might constitute ethical engagement when conducting animal-centered research. However, as discussed below (see Section 5) these approaches are limited in the support they can provide for researchers who, when working with animals, will inevitably face ethically charged and unexpected situations requiring them to deal with the details of the research context and make moment-by-moment decisions influenced by their own ethical position toward the animals they work with.

These limitations came to the fore during a research project, which aimed to investigate the process of designing a technological device for Mobility Assistance Dogs (MADs). In order to assist their human partners, MADs are required to interact with a wide variety of products and interfaces in diverse environments which are human-centric in nature and which fail to meet their user needs (4). Specifically, the research focused on the design of wireless dog-friendly controls that would enable MADs to better assist their human partners while enhancing their own welfare. Designing the dog-friendly controls involved a series of empirical studies (discussed below), during which we worked directly with MADs, their trainers, and human partners, and which raised a series of ethical questions that existing ACI ethics proposals did not address.

These challenges led to the exploration of other ethical frameworks from the discipline of Interaction Design, specifically the field of Participatory Design (5) and the applied ethical approach known as Situated Ethics, which is concerned with how the individual deals with and resolves specific situations – rather than the application of general rules (6). Because animal users are subject to our interpretation of their needs, ethical approaches that stem from an individual frame are of particular relevance for understanding the ethical implications of designing for them. Informed by situated ethical approaches, we developed an ethics toolkit for animal-centered design and research. This comprises three templates that, through a series of questions, assist the researcher in clearly and systematically defining the moral commitments and attitudes that underpin the research. Template A prompts the researcher to articulate their, and the research's ethical baselines as they relate to animals; Template B prompts the researcher to investigate and reflect on how their ethical baselines influence their ethical judgments during animal-centered research; and Template C prompts the researcher to articulate a series of guiding statements to better prepare them to make ethically sound situated decisions during the research. The application of the toolkit during the abovementioned research project with MADs helped the field researcher make ethically sound situated decisions during the research and design of the dog-friendly controls, supporting her interactions with MADs, their trainers, and human partners. It also supported a practice of active reflection in the research team, which proved to be extremely valuable when dealing with unforeseen situations as the research progressed.

This paper discusses the issues that led to the design of the toolkit, the ethics approaches on which it is grounded and how these helped address ethically charged situations during the research. The paper then describes in detail the toolkit components and how to use them, providing an example. It concludes by proposing that the toolkit would provide a valuable resource to support researchers' ethical engagement in any field of research and practice that involves animals.

## The Challenge of Designing Interactive Systems for Animals

While animals have been exposed to interactive technology for decades, for example in precision farming, in conservation efforts or in research settings, a more recent interest on the interactions between animals and technology has led to the development of Animal-Computer Interaction (ACI) (7). As a field, ACI aims at: i. studying and theorizing the interaction between animals and technology in naturalistic settings; ii. developing user-centered technology to improve animal welfare, support animals in their activities and foster interspecies relationships; and iii. informing the development of user-centered approaches to the design of technology intended for animals, enabling them to participate in the design process as legitimate stakeholders and contributors.

To meet these aims, ACI takes a perspective closely aligned with the perspective taken by disciplines such as Human Computer Interaction (1) and Interaction Design (2) which focus on the study, design and evaluation of human-centered interactive systems, with insights from psychology, ergonomics, engineering, informatics, social sciences, product, and service design. This implies a recognition that, to best support people's successful interaction with a system, designers must consider the characteristics and capabilities of those the system is intended for, as well as their activities and the environments in which these activities take place. To this end, during the design process, requirements for a system's usability (i.e., the extent to which the system is easy to use for its intended user) and user experience (i.e., the kind of experience the interaction with the system provides) are elicited from prospective users and other stakeholders to inform alternative designs, which are prototyped and evaluated, in an iterative process of incremental improvement. This process is challenging enough when the stakeholders in question are humans but, when it comes to designing animal-centered interactive systems, the challenges designers face are even greater. These include the potential inability of technology developed by human designers to truly represent the animals' interests; the difficulty of designing from the animal perspective when the barriers represented by interspecies differences, specifically regarding communication, are so significant; the potential for the animal users' interests to be not aligned or even in competition with those of the designers; and the difficulty of interpreting animals' interests without bias (8). Designing for animals requires the development of methods which are “*sufficiently robust but also versatile enough to help deal with the challenges, pitfalls and tensions”* (7) inherent in multispecies interaction design and which can “*reduce the arbitrariness of or biases in choices made during the design process”* (7). In this sense, designing for animals requires a strong ethical commitment toward animal stakeholders, as designers engage *with* them to develop design solutions *for* them; such commitment requires the support of conceptual frameworks and practical tools that can guide designers activities during the process.

## Current Work on Ethics Within Animal-Computer Interaction

Ethics considerations when addressing the challenges involved in designing interactive technology *for* and *with* animals have been an integral part of ACI's concerns from early on, with Mancini (3), first proposing that the discipline's ethical foundation should be based on a non-speciesist relationship between human researchers and animal participants, on the grounds that this would yield more effective interactive systems (3). The author outlined a set of general ethical principles including: preventing any type of discrimination among participants and researchers during the research and design process; protecting all participants from psychological or physiological harm; treating both humans and animals equally during the entirety of the research and design process; considering whether the work being carried out is beneficial and relevant for all participants; affording all participants the ability to withdraw from the research; and enabling informed consent for animal participants. A more detailed set of ethical guidelines was then put forth by Väätäjä and Pesonen (9), which were grounded in the framework of the 3Rs, the most widely recognized standard for humane research. The guidelines highlighted the need to conduct a cost-benefit analysis and to carefully consider animal welfare principles in each phase of any proposed ACI research (9). For example, aspects to consider prior to beginning research, included the justification and prospective benefits of the research, the choice of animal participant, the research procedures and devices, and the training of required personnel. Things to consider during the research included the researcher's responsibility toward the animal, the professional handling and housing of the animal, the approval for the animals' participation, and the monitoring of the animals' well-being. Hirskyj-Douglas and Read (10) also contributed guidelines specifically related to interaction design research conducted with dogs. In addition to ensuring that during research procedures the welfare needs of canine participants were met, the guidelines recommended giving them complete autonomy and even “* avoiding the training of canine participants in the use of technological systems”*, to maintain the research's focus of the dog's *true needs* and to avoid the risk of imposing human requirements on them (10).

Such risk was discussed by Grillaert and Camenzind (11), who highlighted the potential ethical conflicts resulting from the practical application of ACI research, considering the complexity of determining harms and benefits to animal participants vis-à-vis ACI's non-speciesist and welfare-enhancement ambitions. As a way forward, they proposed the use of critical anthropomorphism (11) inviting ACI researchers to use their experience and understanding of the potential harms inherent to humans with technology as a guide to consider ways in which technological interactions might also be harmful to animals. They suggested that an interdisciplinary approach, grounded in animal welfare science, would help ACI researchers to develop ethical frameworks to support the long-term welfare of the animal participant. In this regard, following a critical review of current legislation regulating the use of animals in research, Mancini (7) proposed a research ethics protocol grounded in animal welfare theory, reflecting on the centrality of animals' interests as research partakers and technology users for ACI's non-speciesist approach. The framework covered four fundamental aspects, requiring: that the research be relevant to partaking animals as well as their species; that the welfare, both the integrity and the autonomy, of partaking animals be prioritized over societal interests; that partaking animals, including any humans, be treated impartially regardless of species; that the animals consent be garnered, both from those responsible for their well-being (*mediated consent*) and from the animals themselves (*contingent consent*), through expert monitoring of their behaviors and unconstrained choices during research procedures (8). The importance of being especially attentive to animals needs and wants when designing interactive technology was discussed by French et al. (12) who used speculative design to explore ways in which interspecies communication could be enabled by tech-supported playful activities. The speculative designs prompted a series of reflections related to ethical issues and power dynamics arising between humans and animals during play. The researchers observed how, as the “*top predator(s) in every engagement”* (12), in their interactions with animals, humans have an overwhelming influence irrespective of their intent, such that equitable relationships with non-human animals would be hardly possible. While they argued that ”*humans should start listening a bit more”* (12) as part of their duty of care toward animals, they did not clarify what designers should be listening to or how.

Overall, on the one hand, contributions to the ethical discourse on ACI have provided normative frameworks (e.g., Mancini (3); Väätäjä and Pesonen (9); Hirskyj-Douglas and Read (10), Mancini (8)) for researchers to apply when designing and conducting research involving animals. On the other hand, contributions have cautioned researchers about the challenge of undertaking ACI research with animals without allowing human interests to prevail, exhorting them to carefully consider and manage their own bias (e.g., Grillaert and Camenzind (11), French et al. (12)). However, tools are still lacking which could enable interaction design researchers, who wish to conform to an animal-centered ethics, to identify and manage their own biases during the research process, by reflecting on their own values and on issues that arise as they engage with their animal participants in practice. This paper proposes an ethics toolkit for animal-centered design, which was developed during an ACI research project to address this gap. In the following sections we introduce the project to exemplify some of the challenges that ACI researchers face as they attempt to practice animal-centered research and the issues that arise from these challenges. We then discuss the ethics theories we drew from to develop the proposed ethical toolkit.

## Designing for Mobility Assistance Dogs

The research which led to the development of the toolkit presented in this paper investigated the process of designing a technological device for Mobility Assistance Dogs (MADs). MADs are specially trained dogs who execute tasks on behalf of their human partners, such as those related to self-care, mobility, and other physical activities (13). In assisting their human partners, MADs are required to interact with a wide variety of products and interfaces (e.g., switches, buttons, handles) in diverse environments (e.g., home, public transportation, shops). However, most of the environments and the artifacts the dogs are required to interact with are designed from a human-centered perspective that fails to recognize MADs as legitimate users and therefore fails to meet their usability needs (4). Failing to meet MADs' usability needs does not usually prevent them from being able to assist their partners but it does result in them facing significant challenges that impact their training and working performance, and that ultimately affect their welfare (4) and their experience as technology users, i.e., their user experience (14).

In collaboration with UK Charity Dogs for Good (15), which trains and pairs MADs with people who need their assistance, the research focused on the design of wireless dog-friendly controls that would enable MADs to easily operate domestic appliances such as lamps or kettles, and wired controls that would enable them to easily open motorized doors often found in buildings frequented by the public. The aim was to deliver interfaces that would provide good usability and a good user experience for MADs, thus expediting their learning process during training, enhancing their performance once paired with their assisted human, improving the accessibility of the built environment for both dogs and humans, and supporting the dogs' welfare. Designing the dog-friendly controls involved engaging directly with numerous MADs to understand the dogs' training process and working environment, to elicit their usability requirements consistent with their sensory, cognitive, and physical characteristics, and to evaluate prototypes that we designed based on our grasp of their requirements. This engagement took the form of a series of empirical studies with MADs participants, the design and execution of which raised ethical questions that existing ACI ethics proposals did not address.

For example, the first study carried out was a comparative verification test (16) in which the usability of three existing access controls was tested: a standard issue control and two canine-friendly prototypes (4). During a series of trials MADs and their trainers were asked to open a motorized door by nudging the controls. Because a “nudge” command requires MADs to use their snout to operate the control, one aspect of the control's usability that was of interest to the researcher was how reachable the controls were: 1) at the height recommended by accessibility standards (75 cm); 2) at the height determined by each dog's forward-facing snout (55 cm–65 cm); and the height of a standard electrical socket (to assess the viability of a plug-in control) (45 cm).

Unlike with the controls at a standard height (75 cm) (which required most MADs to jump up, stand on their hind legs, hold their front legs against the wall, while pushing their snout forward to activate the control), reaching the controls at “snout” and “socket” heights did not require physical effort. So, we expected that these would be easier for the MADs to interact with. However, even when interacting with these lower controls, some MADs exhibited signs of frustration (sitting or lying down, low whining, and looking away). Usually, increasing the ease of an interaction decreases user frustration (17), but apparently not in this instance. Beyond being unexpected, these behaviors required the researcher to choose between stopping the trials, to prevent the MADs from experiencing any discomfort, and continuing to pursue the research goal by continuing the trials irrespective of the dogs' frustration.

The researcher knew that frustration is commonly part of dogs' learning process when they progress from familiarization to proficiency. Nevertheless, a determination had to be made regarding the level of discomfort the MADs would be allowed to experience. Was this a level of discomfort that could be expected as part of their learning progression? If so, was this progression in the interest of the dogs to begin with? Was the researcher's assessment of MADs' heightened level of frustration accurate, despite her knowledge of canine behavior? Furthermore, the researcher questioned whether reducing the physical effort for the MADs might increase other kinds of effort. What hidden biases might the researcher have regarding what would be easier or pleasurable for the MADs to interact with? Existing ACI ethics frameworks did not provide the guidance needed to consider this kind of emerging dilemma and inform practical choices as the research progressed.

Another example of circumstances in which the researcher faced unexpected ethical challenges occurred during the second study. This aimed to investigate whether providing MADs with two controls, one to trigger an environmental state (turning on a light) and one to reverse said state (turning off a light) would allow them to connect their actions with the different states (pushing one control makes the environment lighter, pushing another control, makes it darker). To test this hypothesis low fidelity (2) but functional prototypes were constructed. The design consisted of two round push-pads, one blue and one yellow, mounted side by side on a black board and wirelessly controlling a nearby light source. The choice of colors - blue and yellow - was made to help the dogs differentiate the controls against the contrasting background. The prototypes were placed for seven days in three homes where MADs lived with their respective assisted humans. On the first day of the study, the researcher and training staff from Dogs for Good, visited each of the participant's homes. During the visit, the researcher installed the prototypes, and provided detailed instructions of how to test the controls and record observations; and the training staff trained the MADs to use the prototypes and verified that their human partners were able to instruct the dogs to interact with the prototypes as intended.

The prototypes were quickly damaged by the dogs, suffering structural or functional problems that compromised their responsiveness during the study, prompting the human participants to try and fix the prototypes themselves. Furthermore, since the dogs were asked to interact with two separate controls, the single command “*nudge*” to which they were used was no longer usable as it did not distinguish between the two devices. As a result, during the study the human participants continued to ask the researcher for guidance on how to address these issues and, yet again, the researcher faced a complex ethical dilemma, to address which existing ACI ethics guidelines did not provide adequate support. For example, if previously it had been difficult to decide when to stop a trial during which the researcher was present, making the same decision when she was absent and completely reliant on the participants' accounts was now even harder. When should the researcher tell the participants to stop the study? Furthermore, how would she ensure that continuing with the study did not jeopardize the relationship of trust she had developed with the participants, and thus the possibility of conducting future studies with them, given the levels of frustration, disappointment and confusion humans and dogs had experienced? Additionally, could these issues, or the structural and functional fixes implemented by the human participants, have changed the design of the prototypes and resulted in an interaction that might harm the MADs? The fact that participants proactively fixed the prototypes, although appreciated, also raised ethical dilemmas related to their level of involvement in the study. Had the MADs experienced more frustration on account of their partners wanting to fulfill the study requirements?

This kind of ethical challenges occurred commonly during the research. They required the researcher to moment-by-moment weigh-up ACI ethics guidelines, the research's objectives, their knowledge of the animals, and contextual aspects of the studies (e.g., who were the dogs, what humans were present, how many trials had already been observed). Addressing these difficulties, led to the exploration of ethical approaches within interaction design that are used by designers who work with vulnerable populations, including children and differently abled adults (18). The next section discusses these approaches in more detail.

## Situated Ethics Within Interaction Design

Frauenberger et al. (18) argue that interaction design is a quintessentially ethical practice, because it deals with the interface between humans and the products, services, and technology they interact with, meaning that a designer's intent and what they create has a direct impact on users as individuals and as members of society (19). As a practice which entails the active involvement of various stakeholders (e.g., users, designers, other experts), interaction design addresses ethical issues based on three main frames of reference, respectively relating to the professional context, broader society, and the individual (20). Because animals are always subject to our interpretation of their needs, ethical approaches that stem from an individual frame are of particular relevance for understanding the ethical implications of designing for MAD users. Within this frame can be found, for example, approaches from the field of Participatory Design (21), including what is known as situated ethics (20), which requires the designer to cultivate ethical virtues related to the promotion of cooperation between designers, prospective users, and other stakeholders; the empowerment of all participants; and the collective curiosity and creativity of design teams and the stakeholders involved. For Frauenberger et al. (18), each stakeholder is a moral agent, whose participation in decision-making during the design process helps determine the ethical costs and benefits of the technology that is being developed (19). Hence, approaches that help practitioners consider the ethical acts of stakeholders during the design process are especially valuable and, in particular, situated ethics focuses on those aspects of the design process that require researchers to make situated decisions (22). The following sections discuss applied ethical approaches developed within the scope of situated ethics that, given their consideration of situated aspects of empirical research with certain user groups, are also relevant for conducting empirical research with animals.

### Micro-Ethics

Micro-ethics focuses on the seemingly mundane, yet ethically charged exchanges that occur in every interaction between individuals (23). Initially developed for application within health care contexts, the approach has been applied to research within fields as diverse as engineering, computing, and design.

In their work on participatory design with marginalized children, Spiel et al. (23) develop an interpretation of the approach that is particularly relevant for interaction designers who work with MADs, since it focuses on user groups who share some relevant traits with dogs, such as being limited in their verbal and emotional capacity compared to human adults [also recognized by ACI researchers - (24, 25)]. The researchers describe the user-related challenges encountered during two participatory design projects conducted with disabled (allistic and autistic) and visually impaired children; including, the effect of the children's difficulty to manage their emotions during data collection, the influence of the children's caretakers on their behavior during the research, and the children's demonstrations of emotions toward their caretakers and the researchers (26). Being in a position of greater power and control compared to the children, the researchers found themselves having to make moment-by-moment decisions as to how to respond to the children's behavior, which posed serious ethical dilemmas.

For example, one child who had difficulty managing their emotions and was highly reactive to certain situations required the researchers to try to anticipate these reactions and divert the child's attention to other aspects of the design process or to reframe the research experience in a more positive way. In one instance, the researchers did not react in time, and the child violently destroyed parts of the study's technological setup. In response, one of the researchers switched ”*from a playful and collegial approach to a more serious and strict tone … that identified the destruction of the prototype as a point of contention”* (26). Once the child had rejoined the group, he was given the opportunity to voice his needs and, in response, the researchers reframed the incident as an opportunity to test the robustness of the setup. Was the researcher able to strike a good balance between their role, as enablers of the children's free and creative participation in the study, and their duty of care toward all other stakeholders involved? Should the study have been discontinued? If not, should a researcher have been assigned to better anticipate the child's needs or, rather, should the child have been removed from the study?

In another instance, a conflict of intention arose between the child's carer, who wanted the child to learn to interact with one of the study's interactive devices, and the researcher, who was interested in the child's feedback on their spontaneous interactions with the device. The researcher decided to withdraw themselves from the interaction and noted that the carer, in their intent for the child to use the device uttered “*restrictive questions or comments such as ‘no, you're wrong' toward the child”* (26). After a few minutes, the child stopped interacting with the device altogether and moved on to something else. The researchers report that these types of conflicts recurred during the research and that, in order to maintain their relationship with the child's carers, the researchers always opted to withdraw from these interactions altogether. Was this the right thing to do for the children, their carers, or the research? Should a series of rules of engagement with clear consequences and outcomes have been issued prior to beginning the study?

To help researchers deal with challenges such as the ones described above, Spiel et al. (26) propose a framework informed by a *micro-ethical* approach that encourages designers, during the design process, to systematically analyze how their situated ethical decisions might be influenced by their own moral perspectives and ethical frameworks of reference (26). For example, to help negotiate competing values and agendas during the research, they established alternative approaches to working with the children which meant at times ignoring the negative parts of their experience for the benefit of potentially opening up new enriching interactions. They pointed out that doing so made the new interaction “*fleeting and insecure as it can only happen through precariously balancing the values of carers and researchers alike”* (26). The researchers acknowledged that withdrawing from certain interactions to protect their relationship with the child's carers might result in negative experiences for the children and compromise some of the desired conditions for the work. However, the approach seemed more effective in maintaining the carers, and thus the children, more involved during the research.

These challenges and the resulting approaches proved valuable for our project. Indeed, during our own research with MADs, we observed various similarities with the situations described by Spiel et al. (26) during their research with children. In particular, the dogs' expressions of affect, and the way in which they managed their emotions, had an effect on data collection that needed to be dealt with; and the presence of the MADs' familiar trainers and partners during trials had an influence on MADs' behavior and, in turn, influenced the research activities. This resulted in the researcher, trainers, and their partners having to make decisions on behalf of the dogs so that research activities could progress.

For example, during one of the studies in which MADs and their trainers were asked to interact with a more advanced prototype, the researcher had to negotiate when to terminate a trial with one of the MAD's trainers. The prototypes being tested consisted of two main parts, a cylindrical casing that housed the control's electrical components and a rubberized push pad. The aim of the study was to test the usability of the control and the impact on MADs' user experience while interacting with them. The controls came in three diameters; small (9 cm), medium (12 cm), and large (14 cm), and in each size there were controls respectively featuring push pads that traveled different depths, shallow (5 cm) and deep (2 cm). One of the participating MADs had, in previous trials, successfully operated the small and large controls with the shallow-traveling and deep-traveling push-pads, and the small control with the shallow-traveling push-pad. However, when trialing the small control (9 cm) with the deep-traveling push-pad (5 cm), the MAD was visibly struggling to activate the control. The trainer, possibly to give the dog a chance to end the trial with a successful interaction (a practice common in canine training), was urging the dog to persevere. However, from the researcher's perspective, the unsuccessful interaction seemed the result of the cylindrical casing being too small for dog's snout, which prevented him from exerting the force required to activate the deeper-traveling push-pad. Here, the researcher was faced with a choice: let the trial play out and assume the trainer would at some point stop asking the dog to interact with the control; or ask the trainer to stop the trial. Considering that the issue was the ergonomic unsuitability of the control, the researcher asked the trainer to stop the trial; however, she took care of explaining to the trainer that the problem was with the control's design rather than the MAD's behavior or the trainer's handling of the dog.

This decision was ultimately determined by the researcher asking herself a series of questions typical of the micro-ethics approach, such as: “Where is this decision stemming from?” (e.g., *I am going to confidently terminate the trial because the MAD is struggling due to a design issue, which is my responsibility and, thus, the likelihood that my relationship with the trainer will be affected is very low?*); “What personal, group or professional values are guiding the decision?” (e.g., *Is my desire to protect my relationship with the trainer for the sake of future trials affecting the MAD's current experience - would I make the same decision if the issue was not due to the control's design?*); “Would the dog have made a similar decision?” (e.g., *Would the dog have even tried to interact with the control again if not commanded by their trainer?*); “Were the training settings affecting the way the dog approached the control and the force they were able to exert when attempting to operate the control?” (e.g., *Would the dog's interactions with the controls have provided better results if they had been recorded interacting with the controls in more naturalistic settings, rather than during a repetitive controlled trial?*); and “If this decision is guided by a specific set of values, are these values different from other relevant sets of values? If so, how do they differ and why?” (e.g., *As an ACI researcher, I am aiming to obtain rigorous and replicable results in line with the guidelines established by ACI, while the trainer is aiming to build-up the dog's performance in line with the guidelines established by her organization*).

Asking these kinds of questions helped us to consider the ethical implications involved in working with MADs, which arise from the decisions that researchers find themselves making on the dogs' behalf as the work progresses. Asking these questions also helped us to contextualize and assess our decision-making process prompting us to “*reflect on those choices and discuss them, learn from them and improve our capabilities to make ethically sound judgments in the moment”* (26). For example, during the comparative usability study described in section 4, the researcher opted to continue the study by asking herself questions such as “are the heights being tested causing some MADs to be excluded from being able to operate the controls?”, “how much frustration is acceptable for a dog to experience when interacting with a novel object?”, “what is the trainer's feedback regarding the behavior?”, “how do the MAD's previous experiences impact their interaction with the controls?”, “what implications on the research would stopping the study have?”, and “would the dogs opt-out of the trials if they could, or would they be stimulated by the challenge?” As the study progressed, the decision to continue proved to be the right one, as the dogs' apparent frustration lessened when they became more familiar with the controls. However, having considered this kind of questions gave the researcher confidence that her decision-making process had been guided by active ethical reflection.

### Situational and in-action Ethics

Situational and in-action ethics are similar approaches, both of which recognize designers and researchers as active stakeholders during the design process, and both of which regard ethics as a “moving target” requiring the application of design and research methods that leave room for adjustment (19, 22, 27). However, compared to situational ethics, in-action ethics “*shifts the focus from the situated subject to a deeply interwoven and participatory practice”* (19). In their critique of approaches to formal ethics requirements, Munteanu et al. (22) identify what the authors call “ethical triggers”, that is elements that might indicate potential challenges during the research. For example, the researchers reported that, when testing the design of BrailleTouch (28) - a software keyboard for touchscreen mobile phones based on braille typing - their visually impaired participants were so eager to participate in the research that “*They made our goals their own”* (22). This created an ethical tension between the care the researchers had taken to implement ethical principles regarding informed consent and privacy, and the care-less attitudes toward these same principles shown by their participants, who perceived the research “*as less of an experiment and more of a trivial app testing”* (22) and whose desire to contribute to what they perceived as important for their community overrode any privacy considerations. In response, they recommend that researchers develop the ability to assess the unexpected ethical risks encountered during the research and adapt protocols as necessary, to protect the safety, privacy, and dignity of participants, especially those belonging to *vulnerable populations*.

Frauenberger et al. (19) advocate reflection-in-action as the researcher's practice of constantly and actively reflecting on their actions during the research, enabling them to deal with the “*uncertainty, instability and uniqueness”* (19) of the unexpected ethical dilemmas, which might arise and which anticipatory planning may not enable them to deal with. They also highlight how all stakeholders share the responsibility of ethical reflection during the design process. For example, they cite a project whose aim was to apply participatory design approaches *for* and *with* autistic children to create technological artifacts that would enable the children to share their experiences, an activity notoriously challenging for them. The complexity of the research was described as being due to the project's exploratory nature, and to whether the many stakeholders involved (e.g., children, parents, teachers, school administration, special needs pedagogues, and policymakers) shared consistent moral values and how these influenced their responses to the ethical issues that might emerge. Although the team had developed a series of rigorous ethical guidelines prior to the research, these revealed themselves to be skewed toward the perspective of the researchers and not to capture the perspectives of the other stakeholders involved. In response, the team re-engaged with a few of the stakeholders and was able to develop a more nuanced approach. Although this did not entirely reconcile conflicting interests (e.g., the children sometimes expressing their desire to just be ‘normal', while the researchers promoted their neuro-diversity agenda), it nevertheless allowed “*dilemmas to emerge … and be continuously negotiated and checked upon”* (19).

In the case of our project, both the situational and in-action ethics approaches helped us develop a reflective practice throughout the course of the research, from which we were able to draw clear guidance as to how to approach ethically charged situations with the dogs which had not been foreseen and, thus, addressed during the planning stages. For example, a similar situation to the one described by Munteanu et al. (22) emerged during our research as described in section 4, when our human research participants also *made our goals their own* to the extent that they fixed the malfunctioning prototypes themselves. Reflecting on the work of Munteanu et al. (27) prompted us to investigate why this behavior had occurred. When we asked MADs' human partners why they had tried to fix the prototypes, all of them mentioned their interest in being part of a project that would help others like them in their community and improve the lives of MADs. One participant commented how important it was for them to have been chosen to participate in the research and how this made them keen to ensure the study's success by complying with what had been asked of them. All participants mentioned that their MADs had been very frustrated and confused but that eventually, when the device did work, they seemed to be extremely “proud of themselves”. Although it was clear that the MADs' human partners were well-intentioned, their motivations nevertheless raised questions regarding the safety of both humans and dogs (e.g., possible harm caused to either of them due to a malfunctioning prototype), and even the dignity of the canine participants (e.g., having to interact with what was an unusable product). Being mindful of the value that MADs' human partners gave to being part of the research enabled us to take into account how this could impact their participation and the participation they required of their dogs; we also endeavored to distance ourselves emotionally from the design of the controls while engaging with participants, so as to signal that any issues they might encounter with the prototypes during the study would not be taken personally; and we resolved to develop a set of rules of engagement for our next in-home study.

Reflecting on Frauenberger et al.'s. (19) case study with autistic children prompted us to take note of the many stakeholders our research project included (MADs, the researcher, a research fellow, the MAD's trainers and handlers, the MAD's partners, the family members of the MAD's partners, the charity's canine and administrative staff, the project's supervisors, the university, the university's board of ethics, and the project's sponsor) and the ethical risk of producing ethical protocols which failed to capture their perspectives. This risk becomes especially significant when dealing with animal research participants, who are unable to articulate their own ethical perspectives; in turn, prompting the researcher to try and interpret what the animals' perspective might be based on their own assumptions, and their interpretations of the assumptions of the other stakeholders. To mitigate this risk, an alignment meeting attended by the research team, the project's supervisors, and the charity's administrative staff was conducted, during which each stakeholder shared their goals for the project and discussed their expectations regarding the involvement and treatment of the dogs during the research. These discussions provided an open and transparent space to negotiate the moral standpoint of the project's main stakeholders and, thus, revealed a new series of ethical considerations. For example, when the charity's administrators expressed an interest in exposing their employees to the research's progression, new questions emerged, such as “if the dog trainers' involvement in the research is exposed to other members of staff, will the trainers start to interact with MADs any differently?”, “will presenting the project's progress to the organization influence the way the studies are conducted?”, or “will the dogs chosen to participant in the research be regarded and treated by member of staff differently from the ones who are not chosen?”

## An Ethical Toolkit for Animal Centered Research and Design

The approaches and examples discussed above highlight the relevance that situated ethics had when working with MADs and its potential relevance when undertaking interaction design research with animals. To facilitate the application of situated ethics in ACI research and support designers' ethical engagement with animal users and research participants, we developed an ethics toolkit to support animal-centered research and design. The toolkit is the result of weaving together aspects of micro, situational, and in-action ethics that prompt ACI researchers to define what Frauenberger et al. (19) describe as the *project's ethos*, the “*moral commitment or stance, a moral attitude that underlies a particular practice”* (19). Although the toolkit is intended for use by individual researchers, it provides a base for negotiating with other stakeholders the ethical standing of a research project. By prompting them to systematically reflect on their own ethos and perception of the ethos of other stakeholders, the toolkit enables the researcher to acknowledge how their ethical values and perceptions inform their actions, and how their actions influence their values and perceptions in return. The toolkit is designed to support a cyclical reflection process through each new research challenge, so that the ‘ethical profile' of the researcher it is constantly being developed and reflected upon.

Thus, the toolkit aims to help ACI researchers to define their project's ethos clearly and systematically by articulating their and the research's ethical baselines as they relate to animals, to investigate how these influence their ethical judgments during animal-centric research, and to make ethically sound moment-by-moment decisions during the research. Additionally, by encouraging them to actively reflect on the ethical implications of their decisions - both during the research and once their designs are implemented and deployed, the toolkit aims to help researchers to develop their own sensitivity toward the needs of the animals they interact with and to safeguard the animals' welfare. The following section describes the toolkit in detail.

**Figure 1:**
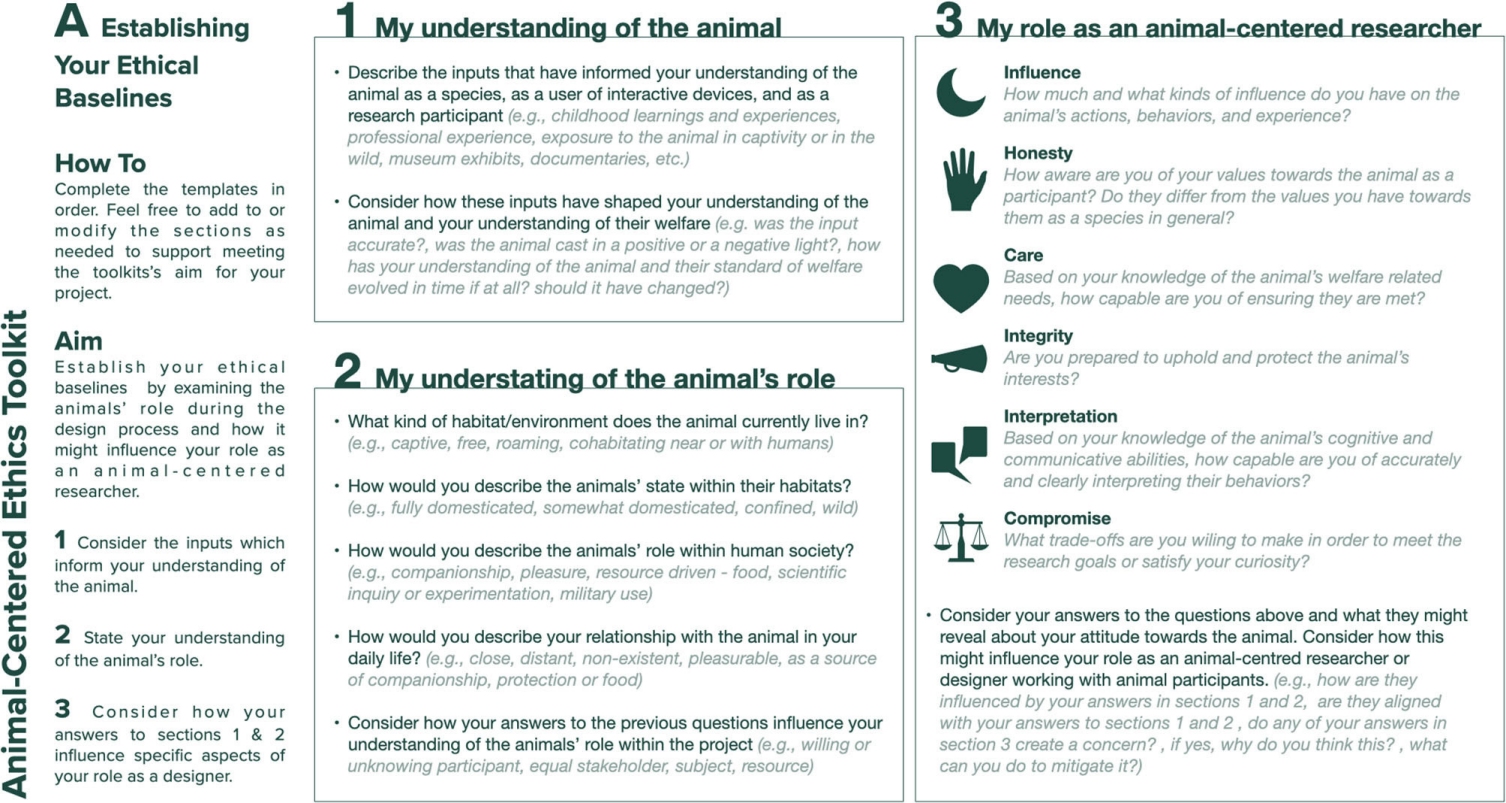
Template A: establishing your ethical baselines.

**Figure 2:**
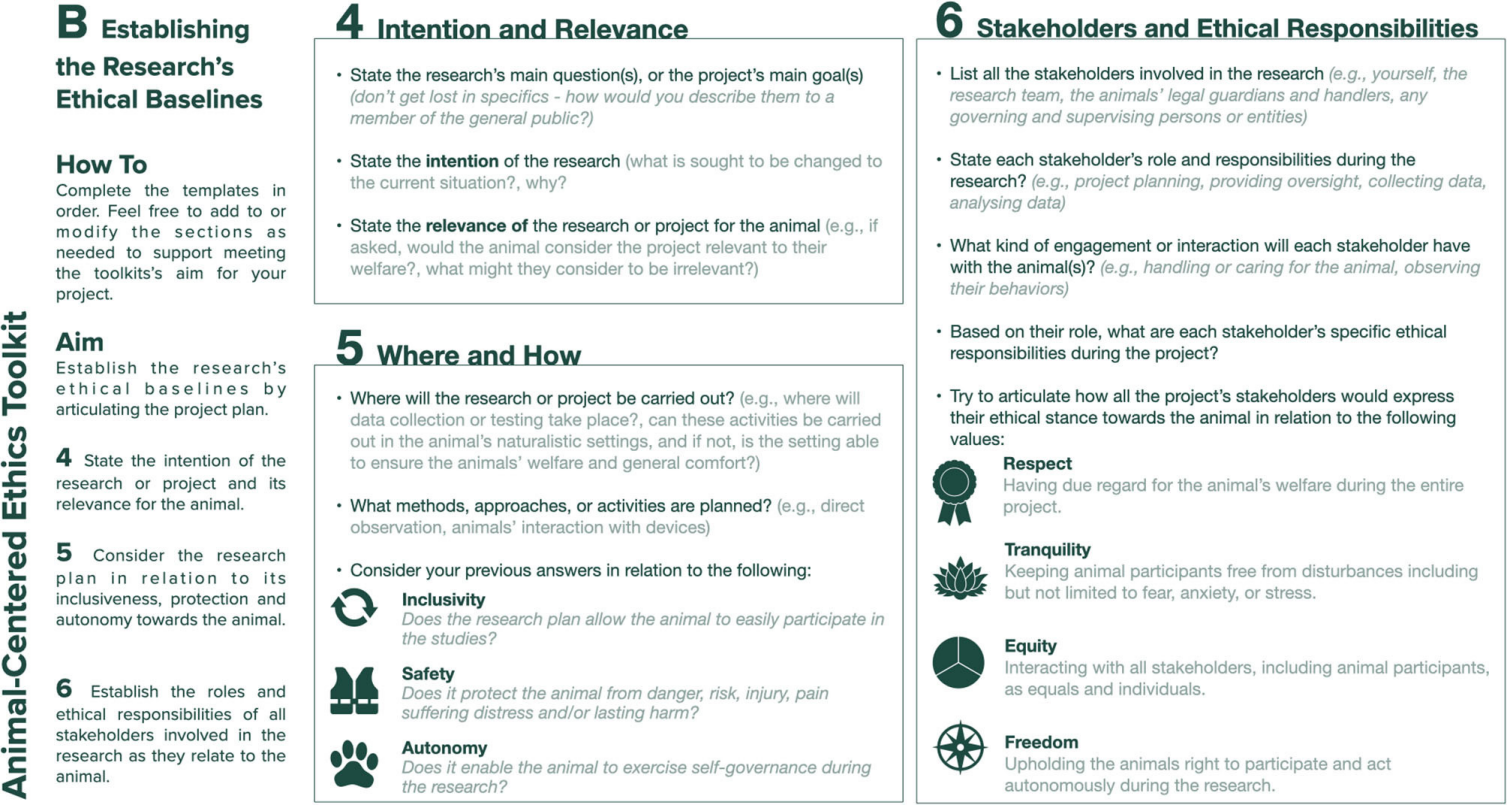
Template B: establishing the research's ethical baselines.

**Figure 3:**
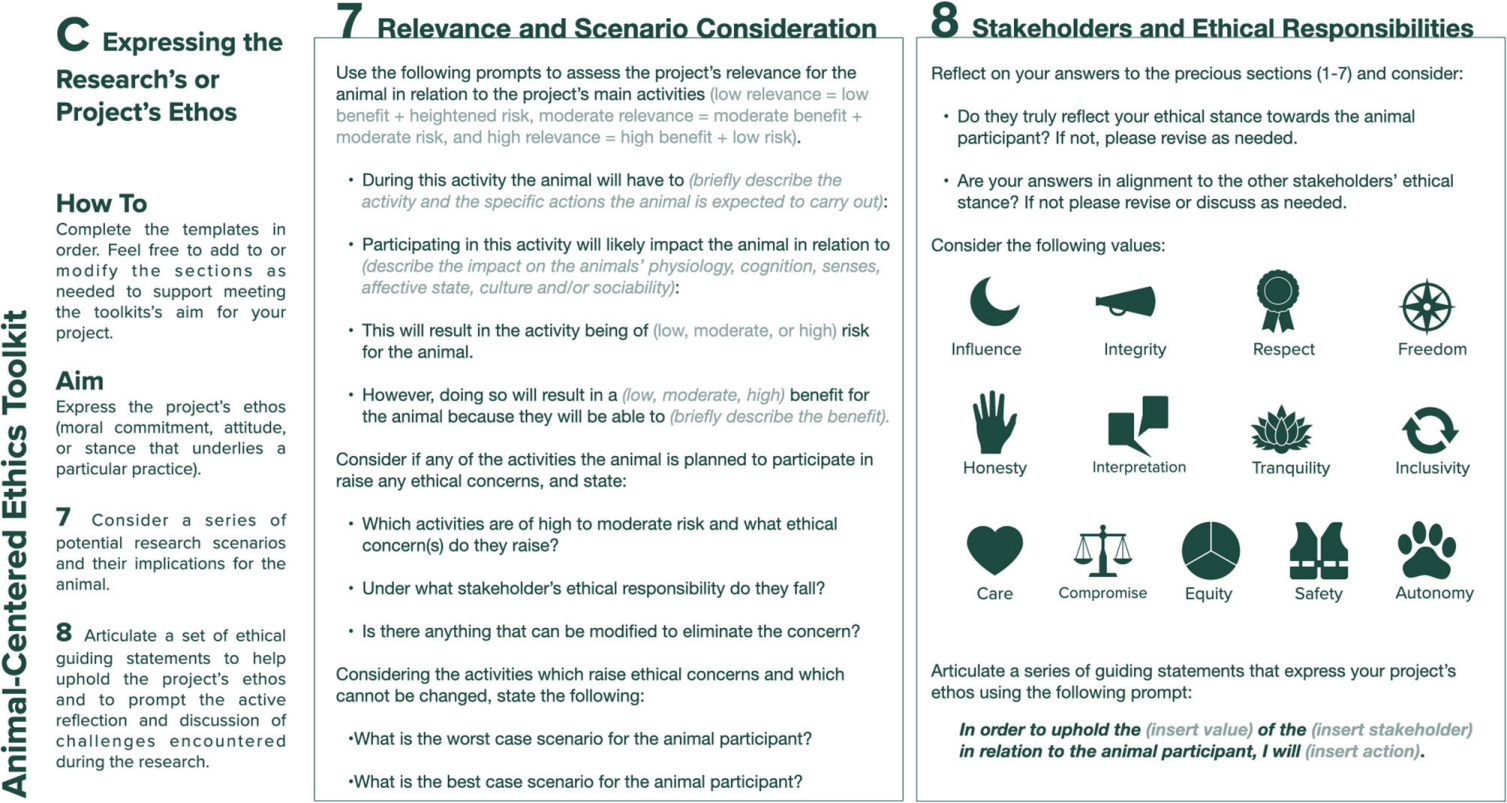
Template C: expressing the project's ethos.

The toolkit is composed of three separate sections shown as separate templates (Figures 1–3), each focusing on a specific aspect of the research:

- Template A: establishing the researcher's ethical baseline
- Template B: establishing the research's ethical baselines
- Template C: expressing the project's ethos.

In the top left-hand corner, each template describes the toolkit's goal, provides an outline of the template's intent, and indicates the steps the researcher will need to take to complete that template. The left side of each template describes the toolkit's steps in more detail, proving researchers with instructions on how to best answer the questions provided. The toolkit is designed to be filled in by individual researchers and designers, to be discussed by the project's stakeholders, actively revised by the project's main researcher or designer, and to be critically reflected upon as a project team at the completion of the research. In the following sections we describe each template in detail, using the terms researcher and designer interchangeably.

### Establishing Your Ethical Baselines (Template A)

Template A's (Figure 1) aim is to help researchers establish their personal ethical baselines by prompting them to carry out a comprehensive assessment of their own understanding of the animal as a research participant and to consider how this understanding might influence their role as an animal-centered researcher. The toolkit's first step asks the designer to consider and reflect upon the inputs that have informed their understanding of the animal as a species, as a user of interactive devices, and as a research participant. Step 2 prompts the designer to consider their understanding of the animal's habitat, the animal's role within human society, their relationship with the animal, and the animal's role within the research. The template then encourages the researcher to reflect on how the combination of these particular aspects might influence their overall view of the animal within the research. The intent of Steps 1 and 2 is to help researchers become aware of the elements that shape their understanding of the animal and to potentially reveal any implicit biases they might have toward the animal, prompting them to question their current beliefs regarding the animal and the animal's welfare. Uncovering such biases is especially important when working with vulnerable user groups (26), animals included, to mitigate ethical challenges inherent in the power imbalance between human researcher and animal participant. To help uncover any inconsistencies that might affect how decisions are made during the research, Step 3 prompts the designer to compare their engagement and relationship with the animal within and outside the research setting. The researcher is invited to consider the influence they have over the animal's actions, behaviors, and overall experience during the research; to honestly consider the alignment of their attitudes toward the animal as a species and as a research participant; to question their ability to care for the animal during the research and to interpret the animals' behaviors, their commitment to protect and uphold the animals' interests, and the compromises or trade-offs they are willing to make. Template A is intended to be used by individual researchers, designers, and the other project stakeholders to capture their personal views.

### Establishing the Research's Ethical Baselines (Template B)

To capture the research's ethical baselines, Template B (Figure 2) shifts the focus of attention from the individual researcher to the research. Step 4 asks the researcher to state the main research question(s) and to consider their intent and the relevance of this for the animal user. Relevance here refers to the balance between the risk and benefit of the research for the animal, based on the principle of “*Doing research that is relevant to participants and consistent with their welfare*” (8) – Toward an animal-centered ethics for Animal Computer Interaction, International Journal of Human Computer Studies, 98p.221–233). Step 5 prompts the designer to state the research's general methodological approach and planned research settings, and to consider how these might impact the animal participant. Specifically, the researcher is invited to reflect on the research activities' inclusivity (how easily the animal will be able to participate), safety (how the researcher will be able to protect the animal from harm) and autonomy (how much self-governance the animal will be able to exercise during the research). Step 6 asks the designer to indicate the project's stakeholders and to consider their roles, responsibilities, and type of involvement (e.g., specific engagement with the animal participant) during the research. It then asks the researcher to clearly articulate stakeholders' ethical responsibilities. The intent behind these questions is to help the researcher to uncover any conflicts or competing goals among project stakeholders in relation to the animal participant so that, when faced whit ethical challenges during the research, conflicts can be more easily articulated and mitigated. The final question in Step 6 prompts the researcher to consider how each stakeholder, including themselves, stand in relation to the values of respect (having due regard for the animal's welfare), tranquility (keeping animal participants free from disturbances including but not limited to fear, anxiety, or stress), equity (interacting with all stakeholders as equals and individuals) and freedom (upholding the animals ability to participate autonomously during the research).

### Expressing the Project's Ethos (Template C)

Template C of the toolkit (Figure 3) comprises Step 7, which prompts the designer to consider a series of research scenarios and their implications for the animal; and Step 8, which helps researchers to clearly articulate a set of ethical guiding statements. The aim of Step 7 is for the researcher to revisit the project's relevance for the animal participant (previously examined in Step 4 of Template B) in light of the information captured in Steps 5 and 6, and their contributions toward achieving a greater awareness of the project's ethos. It prompts the designer to consider if any of the planned research scenarios might raise ethical concerns related to the animal participant not identified during the previous steps; if so, how these might be dealt with or how any aspects of the research plan could be changed or adapted to reduce the concerns. Step 8 invites the researcher to consider their responses to Steps 1–7, and to ask themselves whether they think that the animal participant would have made a similar assessment or whether they need to re-evaluate specific aims or activities in the research plan. It then prompts researchers to consider the project's stakeholders and the values discussed in the previous steps (influence, honesty, care, integrity, interpretation, compromise, inclusivity, safety, autonomy, respect, tranquility, equity, and freedom) and produce a series of ethical statements to help articulate the project's ethos, by responding to the following prompt: *In order to uphold the (insert value) of the (insert stakeholder) in relation to the animal participant, I will (insert action)*. Here, the intent is twofold: firstly, to equip researchers with a series of statements, whose production process will hopefully have helped them unpack the complex, diverse, and nuanced nature of the ethical implications arising during animal-centered research and which enable them to have a critical dialogue “*about the framing, the judgments, the context, and one's own ethical standpoint while responding to ethical dilemmas as they arise*” (19); secondly, to instill a practice of active reflection during the research by prompting a review of their previous responses to the toolkit's questions to ensure that they are aligned with the project's ethical perspectives.

To ensure that the toolkit is consistently updated to reflect the project's ethos in the face of any unexpected challenges or changes due to the practice of active reflection among stakeholders during the research: it is suggested that someone within the team take ownership of the toolkit. Doing so would arguably task said team member with the gathering and recording the team's responses: however, it would provide all stakeholders with a valuable tool to visualize, understand, and act according to the diversity of thought captured in the templates and enrich the definition of the project's ethos.

## The Toolkit in Use - An Example From Designing for Mobility Assistance Dogs

**Figure 4:**
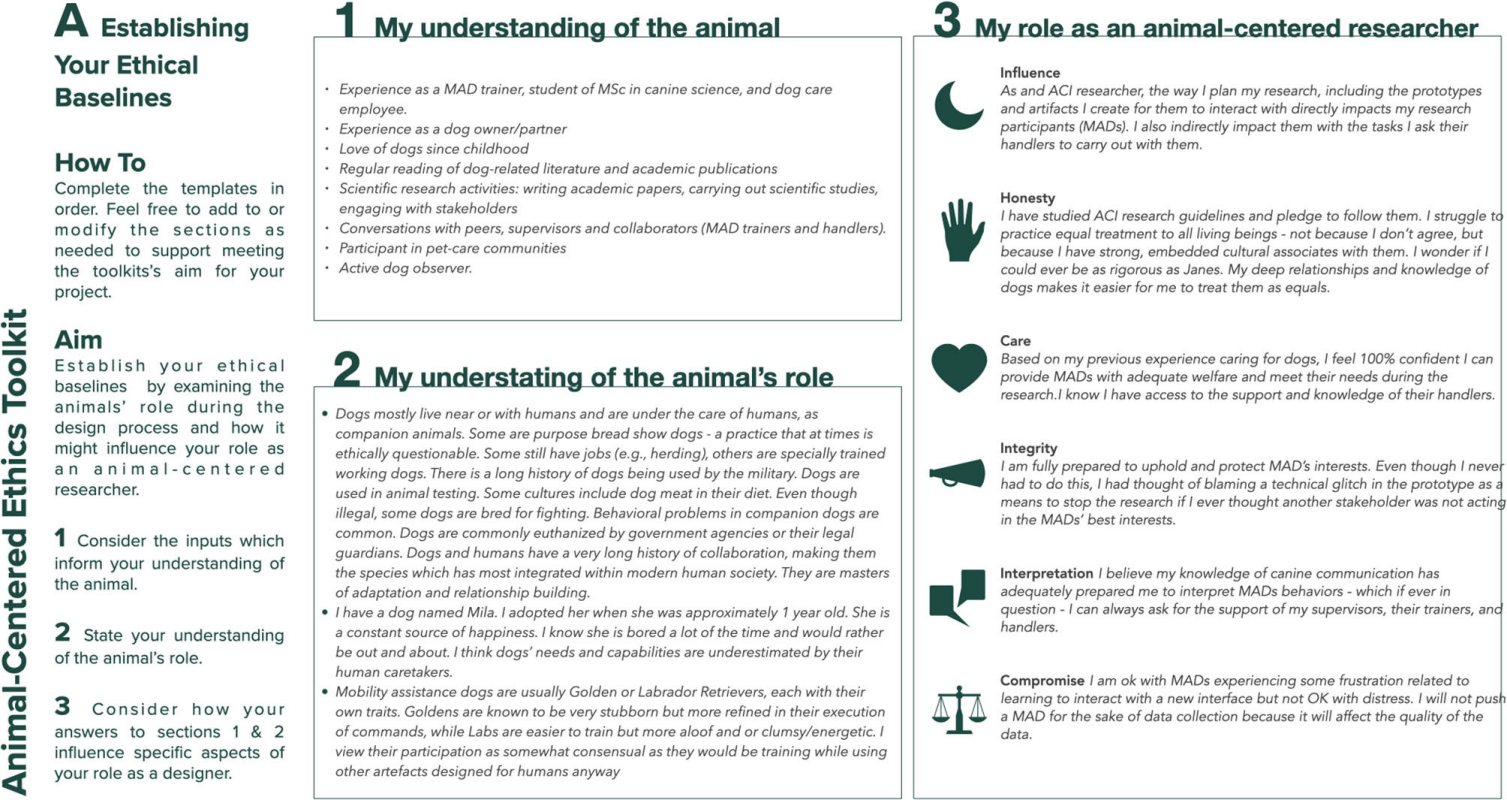
Sample of template A: establishing your ethical baselines for the design of a set of canine-centric controls for Mobility Assistance Dogs.

**Figure 5:**
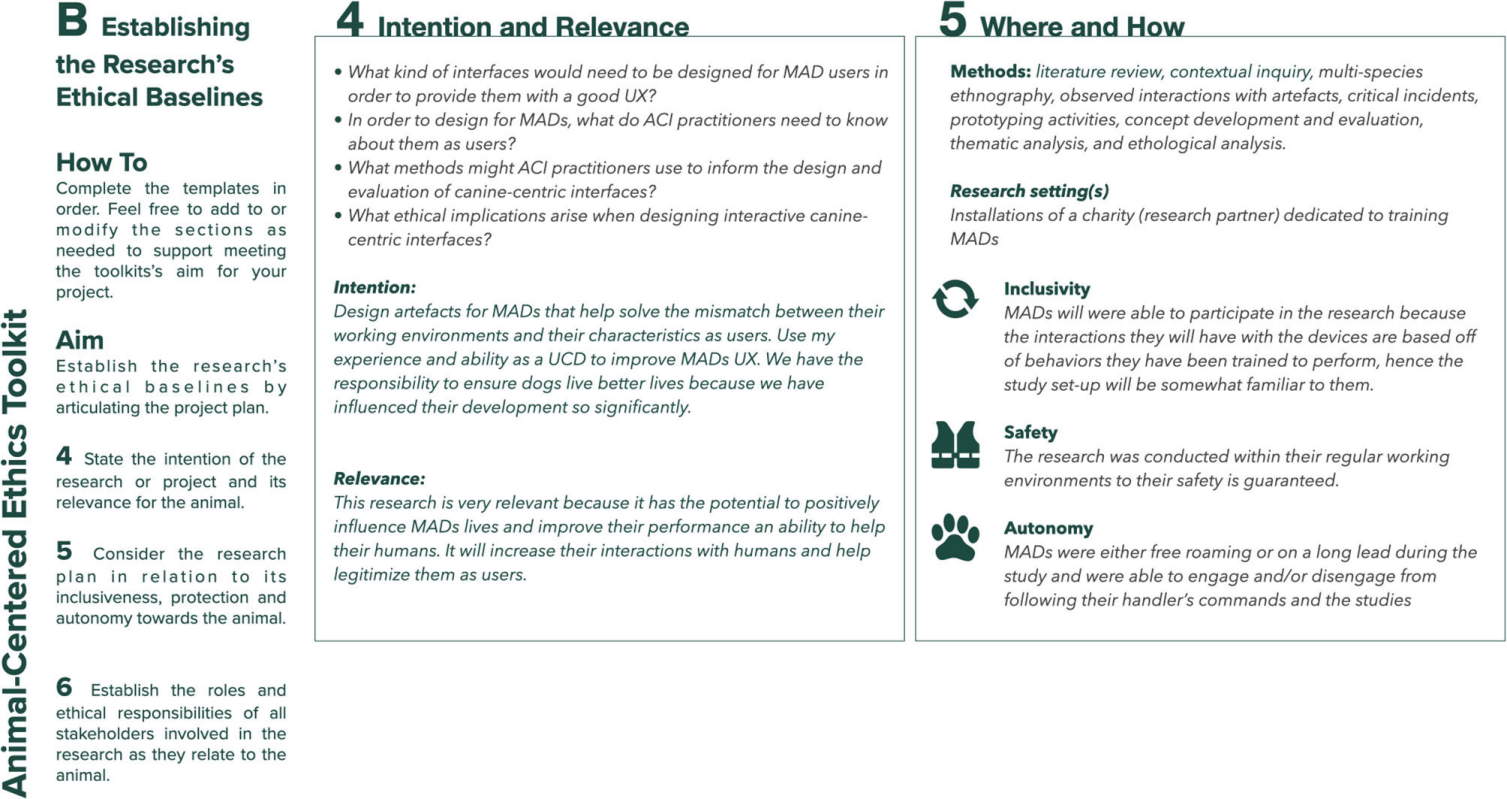
Sample of template B: establishing the research's ethical baselines for the design of a set of canine-centric controls for Mobility Assistance Dogs, steps 4 and 5.

**Figure 6:**
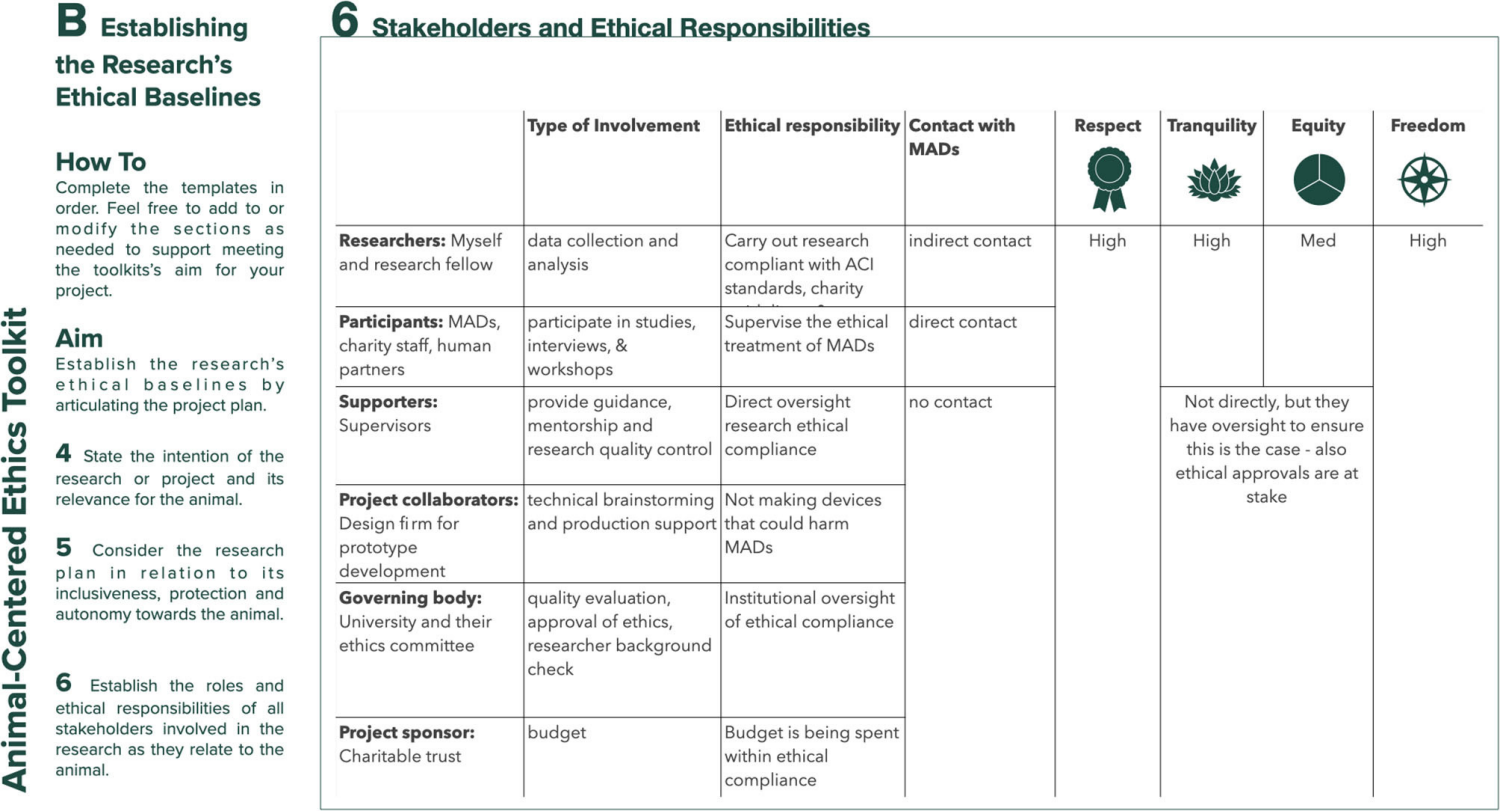
Sample of template B: establishing the research's ethical baselines for the design of a set of canine-centric controls for Mobility Assistance Dogs, step 6.

**Figure 7:**
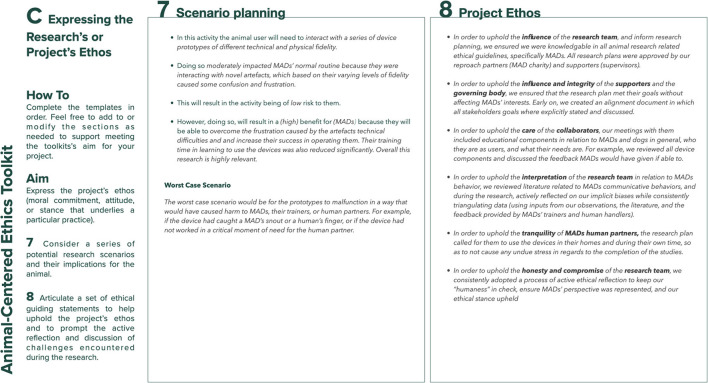
Sample of template C: expressing the project's ethos for the design of a set of canine-centric controls for Mobility Assistance Dogs.

**Figure 8:**
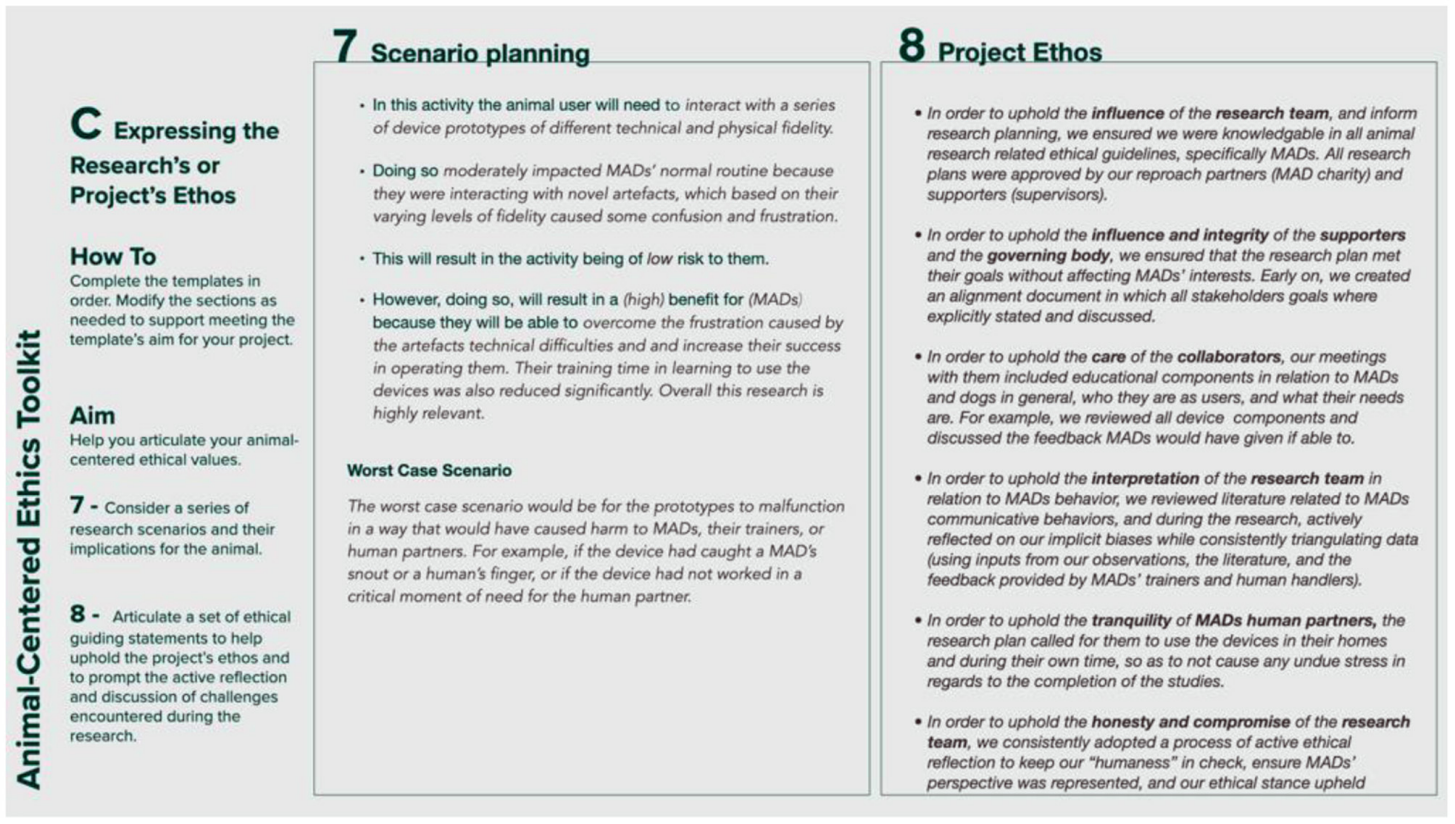
Sample of template C: expressing the project's ethos for the design of a set of canine-centric controls for Mobility Assistance Dogs.

This section provides examples of the type of information the toolkit aims to elicit and how engaging with the toolkit's prompts can help researchers. Figures 4–8 provide a sample of how the project's main researcher made use of the toolkit. For example, completing Template A prompted the researcher to explicitly acknowledge her bias toward typical MAD breeds (Golden Retrievers, Labrador Retrievers, or a mix between them), derived from her previous experience of training MADs (during the research, the same bias was also acknowledged by some of the trainers). Being aware of this bias then helped the researcher to ensure that her expectations on how the dogs might interact with the controls were not influenced by the dogs' breed. For another example, completing Template A also allowed the researcher to acknowledge the difficulty of treating all research participants - both non-human and human - impartially, given the challenges of knowing what the MADs were experiencing and, therefore, of knowing whether they were effectively enabled to express their concerns, just as the human participants were encouraged to do. Template B prompted the researcher to uncover a conflict between the MAD trainers' goals and the goal of the research. MADs training is impacted by a series of factors such as the timing, order, and consistency with which commands are taught. This results in the trainers being extremely focused on making the most of the small training window (no longer than 16 weeks) that they have with the dogs. Consequently, the execution of the studies that involved MADs and their trainers was likely to be influenced by the trainers' goal to make all sessions as productive as possible, while the research's goal was studying MADs' interactions with the controls without the pressure of expected productivity. This awareness then allowed the researcher to adapt the studies' protocol, including longer sessions that allowed the dogs more time to familiarize themselves with the controls at their own pace, providing reliable data for the research and a positive outcome for the trainers. For another example, completing Template C highlighted that, when working with project collaborators such as the design studio tasked with building the controls, the researcher needed to share information about MADs' training, behavior, and working life, in addition to handing over product specifications. This ensured that, during discussions regarding the controls' specific features, everyone had at least a basic understanding of MAD user needs, and their design suggestions were ethically acceptable.

Overall, the toolkit supported the exercise of incrementally building the project's ethos, by fostering an ongoing reflection on aspects of the research that might have been easily taken for granted, enabling the identification of implicit yet influential biases.

## Conclusions: Supporting Ethical Engagement With Animals

Compared to designing for and with humans, designing for and with animals presents an added level of complexity for the researcher, who cannot embody the intended user but nevertheless assumes the responsibility of acting as an interpreter of the animal's behavior throughout the process. In this respect, the role of the ACI researcher is especially demanding, requiring the application of all our powers of observation, empathy, and critical thinking to gain a measure of understanding of the animal as a user and of the elements that may comprise and influence their experience, if not of the experience itself. Aside from scientific competence, this requires ethical sensitivity toward the kind of user interactions and experiences that the animal participant will encounter, the elements of those interactions and experiences that might be important, the way in which these might shift over the course of the research, and the repercussions that such shifts might have.

To this end, the toolkit presented above aims to help animal-centered researchers to clearly and systematically define their projects' ethos by articulating their and their research's ethical baselines in relation to the animals involved; to identify how these baselines might influence their judgments during animal-centered research; and to make ethically sound situated decisions during the research process. Additionally, by encouraging them to actively reflect on the ethical implications of their decisions - both during the research and once their designs are implemented and deployed - the toolkit aims to help researchers to develop their own sensitivity toward the needs of the animals they interact with and to safeguard the animals' welfare.

The toolkit is designed to complement the principles and guidelines provided by animal-centered normative approaches to ethics developed within ACI. While such normative approaches provide essential scaffolding and general guidance for animal-centered research, they are not sufficient to enable researchers to deal with unexpected and ethically charged situations that may arise as the research progresses. By supporting their active and ongoing reflection, the ethics toolkit presented here enables researchers to take a situated ethics approach as they engage with their animal participants. It does so by prompting them to become aware of how their ethical position might influence their research plans, their activities throughout the research, and ultimately their findings. By prompting research teams to articulate a series of guiding statements, the toolkit also helps them develop a shared ethos among team members that is likely to increase compliance. This could provide a common foundation that accounts for multiple ethical dimensions and that can consistently inform decision-making processes, particularly when addressing unforeseen challenges.

The toolkit was developed as a result of the ethical challenges encountered during our research with MADs. Although, in their present form, its constituting templates were designed toward the end of the research process, their composition and the questions that they feature capture the reflection processes that took place as the research was unfolding, informed by a situated ethics approach. As such, we consider the toolkit a live document to be appropriated, modified, and even extended by other researchers as appropriate for their projects, while still maintaining its purpose. In this regard, although the toolkit was developed within the context of Interaction Design and more specifically Animal-Computer Interaction research, we propose that it could support the ethical engagement of researchers who work for or with animals in any other field of research, including within veterinary, welfare and behavioral science. Additionally, we suggest that the toolkit could foster ethical human-animal interactions in any practice settings in which humans work for or with animals or have animal care responsibility, including veterinary practice, specialist training, and even farming.

## Data Availability Statement

The original contributions presented in the study are included in the article/supplementary material, further inquiries can be directed to the corresponding author.

## Ethics Statement

The studies involving human participants were reviewed and approved by HREC The Open University. The patients/participants provided their written informed consent to participate in this study. The animal study was reviewed and approved by AWERB The Open University. During the study, written informed consent was obtained from the dog's owners for the participation of their animals, and mediated consent was obtained from the dogs themselves with the input of their owners and trainers.

## Author Contributions

Based on her prior work on the design of canine-centered controls for Mobility Assistance Dogs, CM contributed to the conception of the research. As the project supervisor, she provided guidance and oversight on the design of studies and compliance with animal welfare and ethics standards. LR, as the main researcher on the project, designed and carried out the studies, conceptualized and created the first draft of the toolkit, and made use of the toolkit during the research. Together, LR and CM revised the toolkit's design and contents. LR wrote the first draft of the manuscript and CM contributed manuscript revisions. LR and CM read and approved the submitted version.

## Funding

This work was supported by PetPlan Charitable Trust.

## Conflict of Interest

The authors declare that the research was conducted in the absence of any commercial or financial relationships that could be construed as a potential conflict of interest.

## Publisher's Note

All claims expressed in this article are solely those of the authors and do not necessarily represent those of their affiliated organizations, or those of the publisher, the editors and the reviewers. Any product that may be evaluated in this article, or claim that may be made by its manufacturer, is not guaranteed or endorsed by the publisher.
